# Supplementary material for: Growth Rate Prediction, Performance, and Biochemical Enhancement of Black Soldier Fly (Hermetia illucens) Fed with Marine By-Products and Co-Products: A Potential Value-Added Resource for Marine Aquafeeds
Source: Insects. 2025 Jan 23;16(2):113. doi: 10.3390/insects16020113 (PMC11855566; doi:10.3390/insects16020113)
Supplement: Supplementary file 1 [file insects-16-00113-s001.zip › insects-3264717-supplementary.pdf]

Supporting information

Growth Rate Prediction, Performance, and Biochemical En-hancement of Black Soldier Fly (*Hermetia illucens*) Fed with Marine By-Products and Co-Products: A Potential Value-Added Resource for Marine Aquafeeds

Daniela P. Rodrigues [1,*], Ricardo Calado [1], Marisa Pinho [2], M. do Rosário Domingues [2,3], José Antonio Vázquez [4,*] and Olga M. C. C. Ameixa [1]

[1] ECOMARE, CESAM—Centre for Environmental and Marine Studies, Department of Biology, University of Aveiro, Santiago University Campus, 3810-193 Aveiro, Portugal; rjcalado@ua.pt (R.C.); olga.ameixa@ua.pt (O.M.C.C.A.)

[2] CESAM—Centre for Environmental and Marine Studies, Department of Chemistry, University of Aveiro, Santiago University Campus, 3810-193 Aveiro, Portugal; marisapinho@ua.pt (M.P.); mrd@ua.pt (M.d.R.D.)

[3] Mass Spectrometry Centre, LAQV REQUIMTE, Department of Chemistry, University of Aveiro, Santiago University Campus, 3810-193 Aveiro, Portugal

[4] Group of Recycling and Valorisation of Waste Materials (REVAL), Marine Research Institute (IIM-CSIC), C/Eduardo Cabello, 6, CP 36208 Vigo, Galicia, Spain

* Correspondence: dmprodrigues@ua.pt (D.P.R.); jvazquez@iim.csic.es (J.A.V.); Tel.: +34-986231930 (ext. 438647) (J.A.V.)

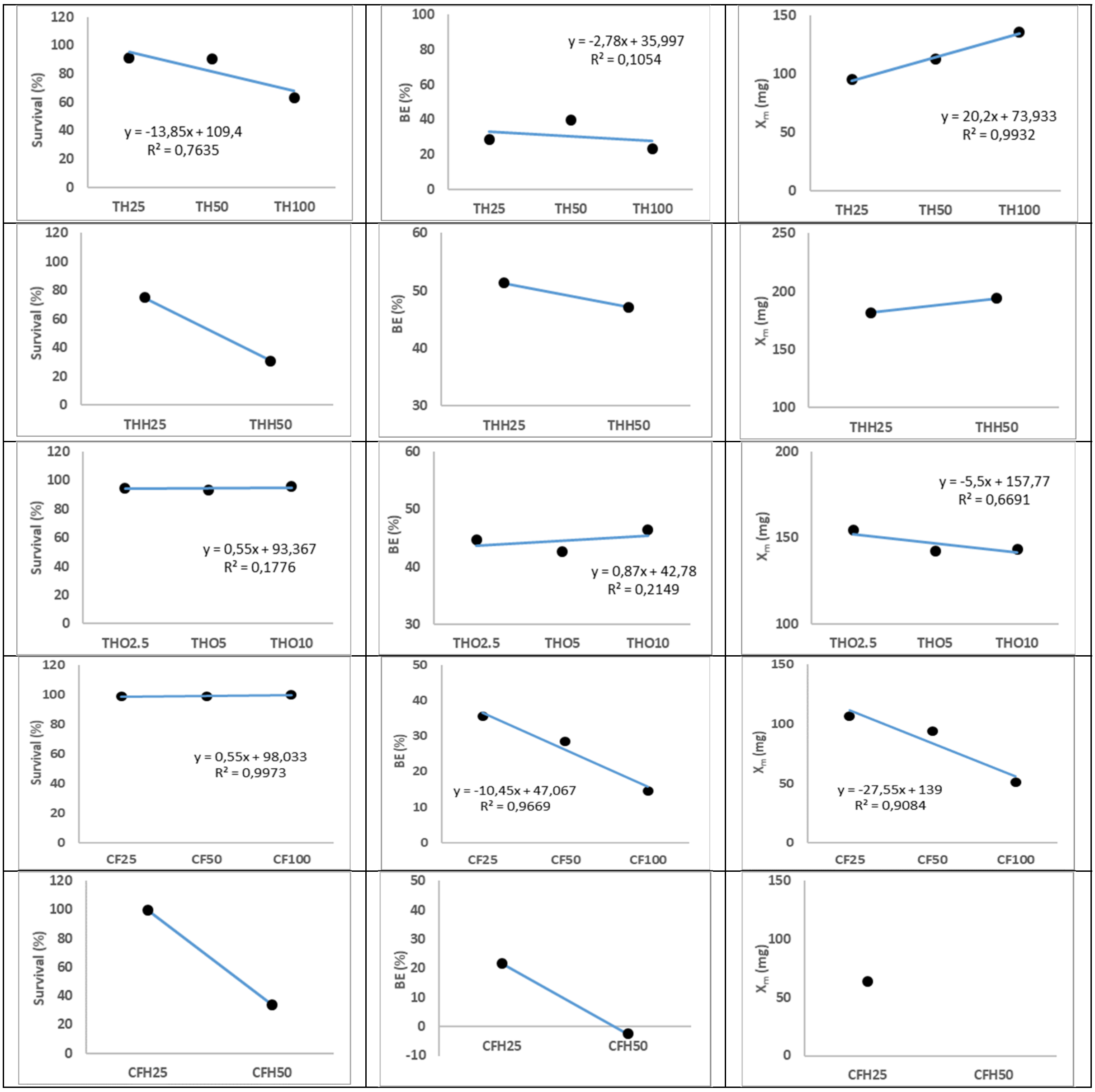

Figure S1. Linear correlation between BSF growth parameters (survival, bioconversion efficiency-BE and maximum biomass-X_m) and the diets provided to BSF with different levels of replacement of tuna heads (TH: untreated tuna heads, THH: tuna head hydrolysates, THO: tuna head oil) and different codfish frames (CF: untreated cod frames, CFH: cod frames hydrolysates). R^2: determination coefficients are also shown.

Table S1. Fatty acids content of the diets provided to black soldier fly larvae (% or g/100 g total fatty acids) with different levels of replacement of tuna heads (TH: untreated tuna heads, THH: tuna head hydrolysates, THO: tuna head oil) and different codfish frames (CF: untreated cod frames, CFH: cod frames hydrolysates). *n*-3/*n*-6 is the ratio as percentage between *n*-3 and *n*-6 fatty acids. Errors are the confidence intervals for n=5 (replicates of processing) and α=0.05.

Trials*	**TH100**	**TH50**	**TH25**	**THH50**	**THH25**	**THO10**	**THO5**	**THO2.5**	**CF100**	**CF50**	**CF25**	**CFH50**	**CFH25**	**C**
**FA 14:0**	2.25±1.63	3.30±0.22	3.47±0.41	2.49±0.34	2.69±0.11	1.96±0.19	1.66±0.08	1.39±0.08	0.99±0.12	0.53±0.14	n.d.	0.46±0.10	0.46±0.10	n.d.
**FA 15:0**	2.86±1.24	2.13±1.17	1.42±0.15	1.24±0.06	1.26±0.01	0.81±0.05	0.70±0.02	0.57±0.02	0.36±0.10	n.d.	n.d.	n.d.	n.d.	n.d.
**FA 16:0**	2.77±1.79	32.75±0.44	35.81±0.08	33.55±0.45	34.55±0.28	19.97±0.22	19.61±0.12	18.73±0.49	21.39±0.73	16.42±0.98	14.61±0.21	16.51±1.01	15.25±0.57	13.30±0.44
**FA 16:1**	5.09±1.29	5.41±1.14	5.91±0.65	6.18±0.55	5.74±0.11	4.74±0.19	4.42±0.39	3.45±0.26	3.96±0.14	1.06±0.36	0.61±0.05	1.07±0.36	0.84±0.29	n.d.
**FA 17:0**	4.36±1.87	3.11±1.36	2.21±0.07	1.23±0.00	1.59±0.57	1.28±0.11	1.12±0.02	0.92±0.04	n.d.	n.d.	n.d.	n.d.	n.d.	n.d.
**FA 17:1**	2.71±1.98	0.93±0.05	0.87±0.06	2.25±0.02	1.77±0.74	0.87±0.32	0.62±0.01	0.48±0.05	n.d.	n.d.	n.d.	n.d.	n.d.	n.d.
**FA 18:0**	10.54±7.17	14.49±0.01	16.98±0.98	15.60±0.26	17.05±0.71	8.49±0.26	8.58±0.19	8.45±0.43	7.08±0.12	6.49±1.14	4.35±0.20	6.53±1.15	5.18±0.13	3.78±0.10
**FA 18:1**	20.93±7.38	26.02±0.19	25.03±0.14	27.18±1.06	25.83±0.59	20.39±0.27	21.05±0.35	21.64±0.77	26.61±1.98	29.21±1.02	32.31±0.18	29.31±1.04	30.76±0.36	33.49±0.60
**FA 18:2**	16.06±14.20	5.30±0.93	2.37±1.27	2.79±0.27	1.50±0.54	6.88±0.17	10.76±0.31	18.17±1.99	1.56±0.29	33.61±4.37	41.09±0.73	33.79±4.35	38.60±5.31	46.90±0.14
**FA 18:3 *n-3***	n.d.	n.d.	n.d.	n.d.	n.d.	0.73±0.08	0.91±0.01	1.38±0.17	n.d.	1.65±0.23	2.05±0.15	1.62±0.28	1.97±0.48	2.52±0.05
**FA 18:4 *n-3***	n.d.	n.d.	n.d.	n.d.	n.d.	n.d.	n.d.	n.d.	1.91±0.12	0.40±0.10	n.d.	n.d.	n.d.	n.d.
**FA 20:0**	0.86±0.01	0.91±0.04	0.83±0.01	0.88±0.02	0.86±0.04	0.48±0.09	0.44±0.01	0.42±0.02	n.d.	n.d.	n.d.	n.d.	n.d.	n.d.
**FA 20:1**	1.98±0.10	1.90±0.14	1.47±0.45	1.73±0.19	1.79±0.10	0.97±0.08	0.98±0.01	0.80±0.02	6.49±0.40	2.03±0.65	1.10±0.14	2.04±0.66	1.43±0.81	n.d.
**FA 20:4 *n-6***	n.d.	n.d.	n.d.	n.d.	n.d.	2.18±0.10	1.87±0.04	1.47±0.07	1.19±0.12	0.76±0.47	n.d.	0.50±0.19	n.d.	n.d.
**FA 20:5 *n-3***	0.71±0.18	0.87±0.05	0.67±0.02	0.69±0.11	0.51±0.17	5.50±0.11	4.74±0.09	3.81±0.02	8.64±1.00	1.88±0.134	1.12±0.15	2.19±0.85	1.42±1.09	n.d.
**FA 24:0**	0.39±0.00	0.46±0.06	n.d.	0.41±0.08	n.d.	n.d.	n.d.	n.d.	n.d.	n.d.	n.d.	n.d.	n.d.	n.d.
**FA 22:6 *n-3***	0.60±0.04	0.68±0.06	0.48±0.05	2.27±1.04	2.86±0.45	24.76±0.83	22.52±0.15	18.3±1.08	19.74±1.6	5.12±1.85	2.76±0.50	5.15±1.86	3.42±2.48	n.d.
**FA 24:1**	2.84±0.10	2.83±0.13	2.46±0.19	1.50±0.70	1.99±0.41	n.d.	n.d.	n.d.	1.62±0.5	0.82±0.34	n.d.	0.82±0.34	0.66±0.16	n.d.
**SFA[a]**	37.68	50.36	51.05	55.40	58.01	32.98	32.11	30.47	29.82	23.45	18.95	23.5	20.89	17.08
**MUFAs[b]**	43.52	36.37	36.51	38.85	37.12	26.98	27.07	26.37	38.68	33.12	34.02	33.2	33.69	33.49
**PUFAs[c]**	18.79	13.27	12.44	5.75	4.87	40.04	40.81	43.15	31.50	43.43	47.03	43.25	45.42	49.42
n-3	7.81	7.97	8.60	2.96	3.37	30.26	27.26	22.13	28.75	7.4	3.88	7.34	4.85	n.d.
n-6	n.d.	n.d.	n.d.	n.d.	n.d.	2.90	2.78	2.85	1.19	2.41	2.05	2.12	1.97±0.48	2.52
**Ratio (*n-3/n-6*)**	n.d.	n.d.	n.d.	n.d.	n.d.	0.10	0.10	0.13	24.12	2.41	0.54	2.94	0.61±0.55	n.d.
